# Processing-induced changes in neuroprotective components and mechanisms of gardeniae fructus: integrating UPLC-Q-TOF-MS/MS, network pharmacology, and* in vitro *analysis

**DOI:** 10.1186/s40643-025-01005-0

**Published:** 2026-01-27

**Authors:** Le Sun, Ziyu Hou, Wenjie Wang, Peiling Wu, Pei Ma, Jiali Huang, Leyang Fan, Lijia Xu, Haibo Liu, Peigen Xiao

**Affiliations:** Institute of Medicinal Plant Development (IMPLAD), Chinese Academy of Medical Sciences and Peking Union Medical College, No. 151 Malianwa North Road, Haidian District, Beijing, People’s Republic of China

**Keywords:** Gardeniae fructus, Gardeniae fructus carbonisatus, Geniposide, Crocetin, Neuroinflammation, Ferroptosis

## Abstract

**Objective:**

Gardeniae Fructus (GF), the dried fruit of *Gardenia jasminoides* J. Ellis, has been used in East Asian medicine for centuries. Its carbonized form, Gardeniae Fructus Carbonisatus (GFC), is produced through processing, yet the effects of this transformation on active constituents and neuroprotective mechanisms remain unclear. This study aims to elucidate the key compositional changes induced by processing and explore their relevance to neuroprotective activity.

**Methods:**

After obtaining GF and GFC extracts via CO₂ supercritical fluid extraction (SFE), UPLC-Q-TOF-MS/MS was employed for qualitative analysis of differential compounds. A pathology-specific network pharmacology screening approach, combined with UPLC-UV-DAD, was applied to quantify major bioactive differential components. Finally,* in vitro* models and molecular pharmacology techniques were utilized to validate the neuroprotective effects of key compounds.

**Results:**

We identified 23 differential compounds and quantified 10 key bioactive constituents. Integrated network pharmacology and quantitative analysis implicated neuroinflammation and ferroptosis in GF’s neuroprotection, with geniposide and crocetin as pivotal compounds. Mechanistic studies confirmed roles for TLR4/NF-κB and Nrf2 pathways.

**Conclusion:**

Geniposide and Crocetin were identified as key compounds responsible for the neuroprotective effects of GF and GFC, primarily through the inhibition of neuroinflammation and ferroptosis. Crocetin is highlighted as a potential marker for GFC.

**Graphical Abstract:**

Processing transforms Gardeniae Fructus into GFC, enhancing glycoside–aglycone conversion and markedly increasing crocetin. Integrated network pharmacology and quantitative analysis reveal geniposide and crocetin as core neuroprotective agents.* In vitro* analysis, these compounds inhibit neuroinflammation and ferroptosis via TLR4/NF-κB suppression and Nrf2 activation, supporting crocetin as a characteristic marker of GFC.

**Supplementary Information:**

The online version contains supplementary material available at 10.1186/s40643-025-01005-0.

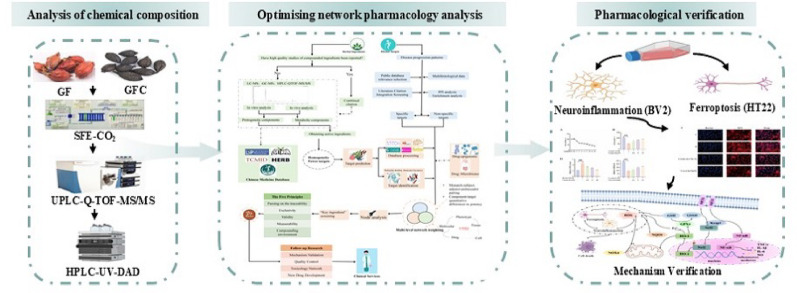

## Introduction

Nervous system diseases (NSDs) constitute a significant global health burden, with their pathogenesis remaining incompletely elucidated (Currais 2015). This broad classification encompasses psychiatric disorders, neurodegenerative conditions, cerebrovascular pathologies, and gliomas - all associated with substantial neurological dysfunction (Bonnechère et al. 2022). Current pharmacotherapeutic strategies predominantly employ receptor modulators and neurotransmitter inhibitors. Although these agents provide symptomatic relief, they exhibit inherent limitations including single-target mechanisms, transient efficacy, high relapse rates, and poor patient adherence, compounded by drug dependency risks and cardiovascular/digestive toxicities, while failing to effectively halt progressive neurodegeneration (Herrmann et al. 2011). With millennia of documented clinical practice, TCM serves as a vital repository of bioactive natural products. Its growing integration into complementary and alternative medicine (CAM) frameworks is particularly noteworthy, with emerging evidence demonstrating distinctive therapeutic potential against NSDs (Guo et al. 2012; Li et al. 2021a).

The pathological mechanisms of NSDs involve multiple processes, including β-amyloid (Aβ) aggregation, hyperphosphorylation of Tau protein, neuroinflammation, and ferroptosis(Jiang et al. 2021; Leng and Edison 2021; Sies 2015; Yuan et al. 2019; Zhang et al. 2021). Network pharmacology, as an evolving systematic research methodology, has gained prominence in deciphering the “multi-component, multi-target” therapeutic mechanisms of Traditional Chinese Medicine (TCM) (Zhang et al. 2023). This interdisciplinary approach enables systematic prediction of bioactive TCM constituents’ potential targets and associated signaling networks through multidimensional data integration (Nogales et al. 2022). Nevertheless, current NSDs-related network pharmacology investigations predominantly adopt a disease-centric orientation, resulting in convergent research perspectives and redundant findings. To address this limitation, contemporary studies are shifting focus toward specific pharmacological axes, exploring TCM’s NSDs intervention potential through mechanism-driven paradigms. This strategic refinement enhances experimental precision and accelerates drug discovery pipelines, thereby bridging traditional empirical knowledge with modern mechanistic validation (Zeng et al. 2022).

Gardeniae Fructus (GF, raw), the dried ripe fruit of *Gardenia jasminoides* Ellis from the Rubiaceae family, is a widely used traditional Chinese medicinal herb (Chinese Pharmacopoeia Commission 2020). Emerging pharmacological evidence highlights the therapeutic potential of GF and its processed formulations in managing neurodegenerative and neuropsychiatric disorders, such as major depressive disorder (MDD), Parkinson’s disease (PD), and Alzheimer’s disease (AD) (Chen et al. 2024; Choi et al. 2024; Zhao et al. 2017). Phytochemical analyses have identified iridoid glycosides (e.g., geniposide) esters as the major bioactive constituents of GF. Using chronic unpredictable mild stress (CUMS) rodent models, Xia et al. revealed that GF-derived iridoid glycosides ameliorate depressive behaviors by potentiating hippocampal synaptic plasticity, mechanistically linked to AMPAR-mTOR axis activation and subsequent upregulation of plasticity-related proteins (Xia et al. 2021). According to pharmacopeial standards, GF is categorized into two primary forms based on processing methods: raw GF (GF) and carbonized GF (Gardeniae Fructus Carbonisatus, GFC). While GF is clinically valued for its heat-clearing and vexation-relieving effects in traditional Chinese medicine (TCM), its potent bitter-cold properties may induce adverse effects such as impairment of spleen-stomach function. In contrast, GFC retains therapeutic efficacy while exhibiting reduced coldness, rendering it more suitable for prolonged clinical use (Zhang et al. 2019; Zhao et al. 2010). This processing-dependent divergence in bioactivity likely stems from distinct phytochemical profiles, though systematic investigations into carbonization processing effects remain limited. Current evidence indicates that carbonization processing primarily alters quantitative chemical composition (e.g., increased crocetin derivatives) without inducing qualitative transformation of constituents (Li et al. 2021a, b). However, the identity of processing-responsive components and their mechanistic contributions to pharmacological outcomes require further elucidation.

Therefore, this study systematically investigates the phytochemical and pharmacological differences between GF and GFC to elucidate their differential neuroprotective mechanisms. We prepared GF and GFC extracts using supercritical CO₂ extraction, followed by comprehensive chemical characterization through UPLC-Q-TOF-MS/MS for compound identification and UPLC-UV-DAD for quantitative analysis. Integrating network pharmacology approaches, we predicted potential molecular targets and pathways associated with Gardenia’s therapeutic effects across various neurodegenerative disease (NSD) pathologies. Subsequent* in vitro* experimental validation confirmed the neuroprotective properties of identified differential constituents. This investigation clarifies the material basis and mechanistic variations in neuroprotection between crude and processed Gardenia, establishing a scientific foundation for optimized clinical application of GFC.

## Materials and methods

### Network pharmacology analysis

#### Bioactive compound detection and target analysis

To systematically identify the bioactive constituents of Gardeniae Fructu*s*, comprehensive data mining was performed using the keyword “Gardeniae Fructus” across several major scientific repositories, including CNKI, Wanfang, PubMed, and SciFinder Scholar. Based on their two-dimensional chemical structures, the Simplified Molecular Input Line Entry System (SMILES) notations for each compound were either manually created using ChemDraw Ultra 8.0 software or obtained from PubChem (https://pubchem.ncbi.nlm.nih.gov/) (Kim et al. 2021).

The HERB repository (http://herb.ac.cn/) (Fang et al. 2021) served to ascertain key constituents in traditional Chinese remedies. Our strategy employed a comprehensive methodology to pinpoint compounds exhibiting favorable pharmacokinetic properties, including oral bioavailability, efficient absorption, strong membrane permeability, and the capacity to cross the blood-brain barrier (BBB). We evaluated candidate compounds in accordance with Lipinski’s Rule of Five (RO5) criteria (Mir et al. 2023). To dig a bit deeper, we ran some pharmacokinetic assessments using the SwissADME platform (http://www.swissadme.ch/) (Daina et al. 2017). We examined essential parameters, including the transdermal penetration factor (Log kp), the logarithm of water solubility (LogS), and the topological polar surface area (TPSA). Only compounds with predicted BBB permeability values of -0.3 or above were retained. The active ingredients were pinpointed by combining insights from UPLC-Q-TOF-MS/MS analysis, literature reviews, and public database resources.

To get a jump on figuring out what GF’s components might be latching onto at the molecular level, we used SwissTarget Prediction (http://www.swisstargetprediction.ch/). We plugged in the SMILES strings and made sure to specify *Homo sapiens* as the species. (Daina et al. 2019). The outputted target data were organized in Microsoft Excel (2019 edition, Microsoft Corp., Redmond, WA, USA) and cross-referenced with disease-associated targets using the Comparative Toxicogenomics Database (CTD, https://ctdbase.org/) and Metascape (http://metascape.org). Only diseases associated with statistically significant targets (*p* < 0.05) were considered. Overlapping targets between the compound-related and disease-related sets were identified, and visual representation of the data was generated using GraphPad Prism 8.0.

To evaluate compound safety, the ProTox-II online tool was employed to predict toxicological characteristics such as acute oral toxicity (LD50, mg/kg), mutagenic potential, immunotoxicity, carcinogenicity, and cytotoxic effects (Oner et al. 2023). Compounds that fell into toxicity category 3 or higher and exhibited more than three toxicity endpoints were flagged as potentially hazardous (Wei et al. 2021).

#### Protein-protein interaction (ppi) network assembly and functional enrichment

The STRING resource (https://string-db.org/) aided in building a protein interaction network to investigate possible GF protein target interrelations (Szklarczyk et al. 2025). The interaction data we gathered was put to good use by constructing a comprehensive network that connected bioactive compounds, therapeutic targets, and the connections to diseases, all facilitated by the Cytoscape software, specifically version 3.7.1. The selected targets removed from the network underwent further investigation through Metascape’s enrichment analysis, incorporating both Gene Ontology (GO) classifications and KEGG pathway examination (Zhou et al. 2019).

#### Identification of differential compounds associated with nsd pathogenesis

This investigation prioritized the Alzheimer’s disease pathway (KEGG ID: hsa05010) to determine which GF-derived components might influence key molecular players involved in amyloid-beta (Aβ) metabolism(Zeng et al. 2021). Critical regulatory proteins such as APP, α-, β-, and γ-secretases, along with Aβ-degrading enzymes like NEP, MME, and IDE, were analyzed. GO term clustering and an extensive literature survey were utilized to uncover targets and corresponding molecules related to Tau hyperphosphorylation and neuroinflammatory cascades. Additionally, ferroptosis-related genes were collected from the FerrDb database (http://www.zhounan.org/ferrdb/current/), allowing for overlap analysis between GF-associated targets and genes implicated in ferroptotic cell death (Yu et al. 2012).

#### Validating key molecular binding via docking

To determine binding aptitude, AutoDock Vina facilitated molecular docking for specified phytochemical-protein pairs (Gouthami et al. 2022). Three-dimensional conformations of the ligands were either retrieved from PubChem or constructed using ChemDraw and Chem3D tools. Crystal structures of receptor proteins, with resolution better than 2.5 Å, were sourced from the RCSB Protein Data Bank (http://www.rcsb.org/). Binding energies (ΔG, kcal/mol) were calculated, with values equal to or below − 7.0 kcal/mol suggesting strong ligand-receptor interactions (Goodsell and Burley 2020). The docking poses and intermolecular contacts were visualized using PyMOL software.

### Reagents and chemical materials

The GF used in this study was sourced from Beijing Tongrentang (Beijing, China). Its identity was taxonomically confirmed as the dried fruit of *Gardenia jasminoides* Ellis by the corresponding author, Professor Haibo Liu. To prepare GFC, the crude herb underwent stir-frying at a temperature of 240 °C until the outer layer became charred black while the inner part exhibited a brown coloration, aligning with traditional carbonization protocols.

A panel of ten high-purity phytochemical standards (≥ 98%) was obtained from Chengdu Aifa Biotechnology Co., Ltd. (Chengdu, China). These included 3-Methylkaempferol (AFCE2202), Gardenin B (AFCL2654), GBGB (AFBG0602), Geniposide (AFDG1956), Genipinic acid (AF21041103), Genipin (AFDA0253), Crocetin (AFCD1712), and Crocins I–III (AFDF1851, AFBG1331, AFCE2203). High-performance liquid chromatography (HPLC)-grade acetonitrile (≥ 99.9%) and formic acid (≥ 99.9%) were purchased from Fisher Scientific (Pittsburgh, PA, USA) and ANPEL Laboratory Technologies (Shanghai, China), respectively. All other chemical reagents used in the experiments met a minimum purity standard of 98% and were supplied by Beijing Chemical Works (Beijing, China). Ultrapure water utilized in the experiments was produced using a Milli-Q purification unit (Millipore, USA).

Reagents for cell-based assays included dimethyl sulfoxide (DMSO, culture grade) purchased from Beijing Sunshine Biotechnology Co., Ltd. Dulbecco’s Modified Eagle Medium (DMEM) and phosphate-buffered saline (PBS) were provided by Nanjing Biotunnel Biotechnology Co., Ltd., while fetal bovine serum (FBS) was acquired from Procell Life Science & Technology (Wuhan, China). Antibiotics (penicillin–streptomycin solution) were sourced from Gibco (USA). Lipopolysaccharide (LPS, DH183-1) was obtained from Beijing Dingguo Changsheng Biotechnology Co., Ltd., and erastin was purchased through Fisher Scientific (USA).

Cell viability tests were conducted using the Cell Counting Kit-8 (CCK-8, APExBIO, Houston, USA). The Griess reagent used to measure nitrite was supplied by Beyotime Biotechnology (S0021, Shanghai, China). The enzyme-linked immunosorbent assay (ELISA) kits required to identify BCA, ROS, and cytokines such TNF-α, IL-1β, IL-6, IL-4, IL-10, and IL-22 were supplied by Beyotime Biotechnology (Nanjing, China). The test kits for malondialdehyde (MDA) and reduced glutathione (GSH) were acquired from Nanjing Jiancheng Bioengineering Institute (A003-4-1, Nanjing, China).

TransGen Biotech (Beijing, China) provided the PerfectStart^®^ Green qPCR SuperMix, TransScript One-Step gDNA Removal and cDNA Synthesis Kit, and TransZol Up reagent for the molecular analysis. Radioimmunoprecipitation Assay (RIPA) buffer (CWBIO, China) was used for protein lysis, and Beyotime Biotechnology (Shanghai, China) supplied the phenylmethylsulfonyl fluoride (PMSF).

For Western blot analysis, the following primary antibodies were employed at specified dilutions: GPX4 (1:5000, ab252833), HO-1 (1:2000, ab189491), and NQO1 (1:5000, ab80588) sourced from Abcam (USA). Additionally, TLR4 (1:1000, #14358), NF-κB p65 (1:1000, #8242), phospho-NF-κB p65 (1:1000, #3033), Keap1 (1:1000, #8047), and Nrf2 (1:1000, #20733) were obtained from Cell Signaling Technology (USA). Horseradish peroxidase (HRP)-conjugated secondary antibodies (1:5000) were procured from CWBIO (China).

### Sample and standard solution preparation

The dried GF and GFC samples were initially pulverized into a fine powder using a lab grinder, then passed through a 40-mesh sieve to ensure uniformity. Exactly 14.00 g of the sieved material were then carefully transferred into a 50 mL extraction vessel for further processing. Using a Waters system (USA), supercritical fluid extraction (SFE) was carried out under ideal circumstances, The parameters included maintaining the extraction temperature at 40 °C, applying a pressure of 200 bar, and setting the CO₂ flow rate to 0.25 mL/min over a duration of 100 min. To guarantee total elimination of any remaining organic solvents, the co-solvent was removed as soon as possible using a nitrogen evaporator (Shanghai Zigui Instrument Co., Ltd., China). After that, the extracted material was kept at -20 °C until it was needed again.

The desiccated product was reconstituted using ethyl acetate to a 5 mL end volume. Next, we used methanol to dilute 0.2 mL of this combination ten times. After passing through a 0.22 µM nylon filter, it was introduced into the UPLC-Q-TOF-MS/MS apparatus. We made a 100 mg/mL solution by dissolving the freeze-dried extract in DMSO for the* in vitro* testing. Using a 0.22 µM filter, we cleaned this before adjusting the concentration to the proper amounts for the cell culture medium.

To prepare the standard solutions, we meticulously weighed ten specific phytochemicals, ensuring their accuracy, and dissolved them in a 70% methanolic solution that was predominantly aqueous. Stock solutions with a concentration of 1 mg/mL were produced as a result of this procedure. These stock solutions were then purified by being filtered through 0.22-µm nylon membranes, which effectively extracted any particulates. They were then ready for the UPLC-Q-TOF-MS/MS analysis.

For* in vitro* validation, standards were prepared in DMSO to achieve a final concentration of 100 mM. We again subjected these solutions to filtration via 0.22-µm sterile filters. Just before conducting the biological assays, each solution was meticulously diluted to the appropriate concentration using the culture medium.

### UPLC-QTOF-MS/MS analysis

A Waters ACQUITY UPLC CLASS system equipped with a BEH C18 column (2.1 mm × 100 mm, 1.7 μm particle size) was used to do the metabolite profiling. Mobile phases A (acetonitrile) and B (0.1% formic acid solution in water) were used in a gradient elution procedure. The following is how the elution profile was programmed: 5% B for 0–2 min; 5–15% B for 2–5 min; 15–50% B for 5–18 min; 50–95% B for 18–25 min; 95 − 5% B for 25–28 min; and 5% B for 28–35 min. Flow rate: 0.3 mL/min; column temperature: 30 °C; 2 µL sample injected per run.

HPLC-Q-TOF-MS/MS (SYNAPT G2-Si, Waters, USA) was used for mass analysis in both positive and negative ionization modes. Key instrument parameters included a capillary voltage set at 2.0 kV, a cone voltage of 40 V, and a desolvation gas heated to 300 °C flowing at 900 L/h. Additionally, the cone gas was maintained at 50 L/h and 100 °C. MassLynx version 4.1 handled data capture and analysis across a 100–1200 m/z range.

To streamline the identification of compounds, a curated in-house database comprising 217 Gardenia-related molecules (with corresponding molecular formulae, exact masses, and SMILES structures) was imported into the Progenesis QI software. The processing workflow involved chromatographic alignment, feature extraction, normalization, and deconvolution, referencing a mixed standards chromatogram.

Features with a coefficient of variation (CV) $\:\le\:$ 0.2 were selected for further screening. Statistically significant markers (VIP > 1 and *p* < 0.05) were identified through multivariate analyses using SIMCA software, including both PCA and OPLS-DA models.

### Quantification of differential metabolites

Using an Agilent Eclipse Plus C18 column and an HPLC-UV-DAD system (Thermo Fisher Ultimate 3000 UPLC), a targeted quantitative assessment of the differential ingredients was carried out. Mobile phases A (0.1% formic acid in water) and B (acetonitrile) of a binary solvent system were employed for the separation process. The following was the gradient elution program: 0–3 min at 5% B; 3–8 min at 5–40% B; 8–12 min at 40–50% B; 12–16 min at 50–65% B; 16–20 min at 65–75% B; 20–28 min at 75–85% B; 24–28 min at 5% B; and 28–35 min at 5% B for equilibration. Using a 2 µL injection volume and 0.3 mL/min mobile phase flow, the column was maintained at 30 °C.

Detection was performed at multiple wavelengths (238, 254, 370, and 440 nm) using a diode array detector (DAD) to capture analytes with varying UV absorption profiles.

### *In vitro* experimental validation

#### Cell culture

The HT22 hippocampal neurons and BV2 microglial cells, both of murine origin, were generously supplied by the Cell Resource Center at IBMS, CAMS/PUMC in China. The cells were nurtured in DMEM with a 10% kick of fetal bovine serum, plus a mixed bag of antibiotics that included 100 U/mL penicillin and 100 µg/mL streptomycin. They were kept cozy at 37 °C, with a 5% whiff of CO₂ in a nice and toasty incubator, all to keep them thriving in prime condition.

#### Cell viability assay

BV2 cells (5 × 10³/well) in 96-well plates were treated with different extract/compound doses for 3 h, followed by 2 h co-incubation with 1 µg/mL LPS. The neuronal HT22 cell model was prepared by plating 2.5 × 10⁴ cells per well, pretreating them with test chemicals for 3 h, and then co-incubating them for 24 h with 10 µM Erastin. To ascertain cell vitality, we resorted to the CCK-8 technique. Once the culture medium was discarded and the cells were thoroughly rinsed with PBS twice, we dispensed a judicious 100 µL of the pre-mixed CCK-8 solution (a concentration of 10% in DMEM) into every well. Post incubation at the sweltering 37 °C for a spell of two hours, we meticulously quantified the optical density at the wavelengths of 450 nm using the Swiss-engineered Tecan microplate reader. Each treatment group was conducted in triplicate with three independent biological replicates.

#### Griess assay

BV2 cells were initially exposed to test compounds for 2 h, after which lipopolysaccharide (LPS, 1 µg/mL) was introduced for a subsequent 24 h stimulation. Post-treatment, culture supernatants (50 µL) were harvested and transferred into a 96-well plate, where they were combined with equal volumes of Griess reagents I and II to initiate colorimetric detection of nitric oxide (NO). Absorbance was recorded at 540 nm using a multimode microplate reader. All experimental conditions were conducted in triplicate with three independent biological replicates.

#### ELISA analysis

After 3 h of exposure to test compounds, BV2 cells were stimulated for a further 24 h with lipopolysaccharide (1 µg/mL). After treatment, cells were collected, and the total protein level was determined by BCA method. The cytokine levels in the culture supernatants, such as TNF-α, IL-1β, IL-6, IL-4, IL-10, and IL-22, were then measured using ELISA kits. For every condition, three biological duplicates of each experiment were performed.

#### Measurement of MDA and GSH levels

After 3 h of exposure to test compounds, HT22 cells were exposed to 10 µM Erastin for 24 h. After treatment, cells were collected, and the total protein level was determined by BCA method. The levels of MDA and GSH were quantified according to the manufacturer’s instructions. Each group of experiments had three biological replicates.

#### Assessing cellular ROS levels

HT22 cells were seeded in 6-well plates and treated with 10 µM Erastin, with or without the compounds. To assess intracellular ROS levels, cells were incubated with the fluorescent probe DCFH-DA, diluted 1:1000 in DMEM, for 20 min. After incubation, cells underwent three PBS washes. Fluorescence recording utilized an inverted microscope (MF52-N, Mshot, China). Emission was measured at 525 nm, while excitation was set at 488 nm. Fluorescence intensity was measured using ImageJ software (National Institutes of Health, USA). For every experimental condition, three biological replicates were conducted.

#### Quantitative real-time PCR (qRT-PCR)

Total RNA was extracted from HT22 cells using the TransZol Up reagent. Utilizing a NanoDrop ND-1000 spectrophotometer (Implen, USA), the RNA integrity and concentration were assessed. The TransScript One-Step gDNA Removal and cDNA Synthesis Kit was then used to reverse transcribe 500 ng of RNA into complementary DNA (cDNA). The PerfectStart^®^ Green qPCR SuperMix Kit and particular primers for each gene were used in the amplification process, which was conducted using a Bio-Rad CFX96 Touch PCR machine (Bio-Rad, USA). The Supplementary Table contains a list of the primer sequences. Using the 2^−ΔΔCt^ technique, relative gene expression levels were ascertained.

#### Western blot analysis

To stop protease activity, HT22 cells were isolated and lysed in RIPA buffer enhanced with PMSF. The total protein level was determined by BCA method. 20 µg protein samples underwent separation via 8% SDS-PAGE, followed by transfer to PVDF membranes(Millipore, USA). The membranes were then blocked using 5% skim milk solution and subsequently washed with TBST buffer. The membranes were treated with primary antibodies specific to TLR4, NF-κB p65, Phospho-NF-κB p65, Keap1, Nrf2, GPX4, HO-1, and NQO1 for overnight at 4 °C. HRP-conjugated secondary antibodies (1:5000, CWBIO) were subsequently applied to the membranes. Protein bands were detected using an ECL kit and imaged with a Bio-Rad system (Bio-Rad Laboratories, USA).

### Statistical analysis

The research was conducted utilizing GraphPad Prism 8.0 software from GraphPad Software in the United States. To determine if there were any statistical variations, we employed a one-way ANOVA, followed by Tukey’s test for pairwise comparisons. We deemed any p-value below 0.05 to be indicative of a statistically significant difference.

## Results

### Screening of active compounds in gardenia with potential Anti-NSDs effects

**Fig. 1:**
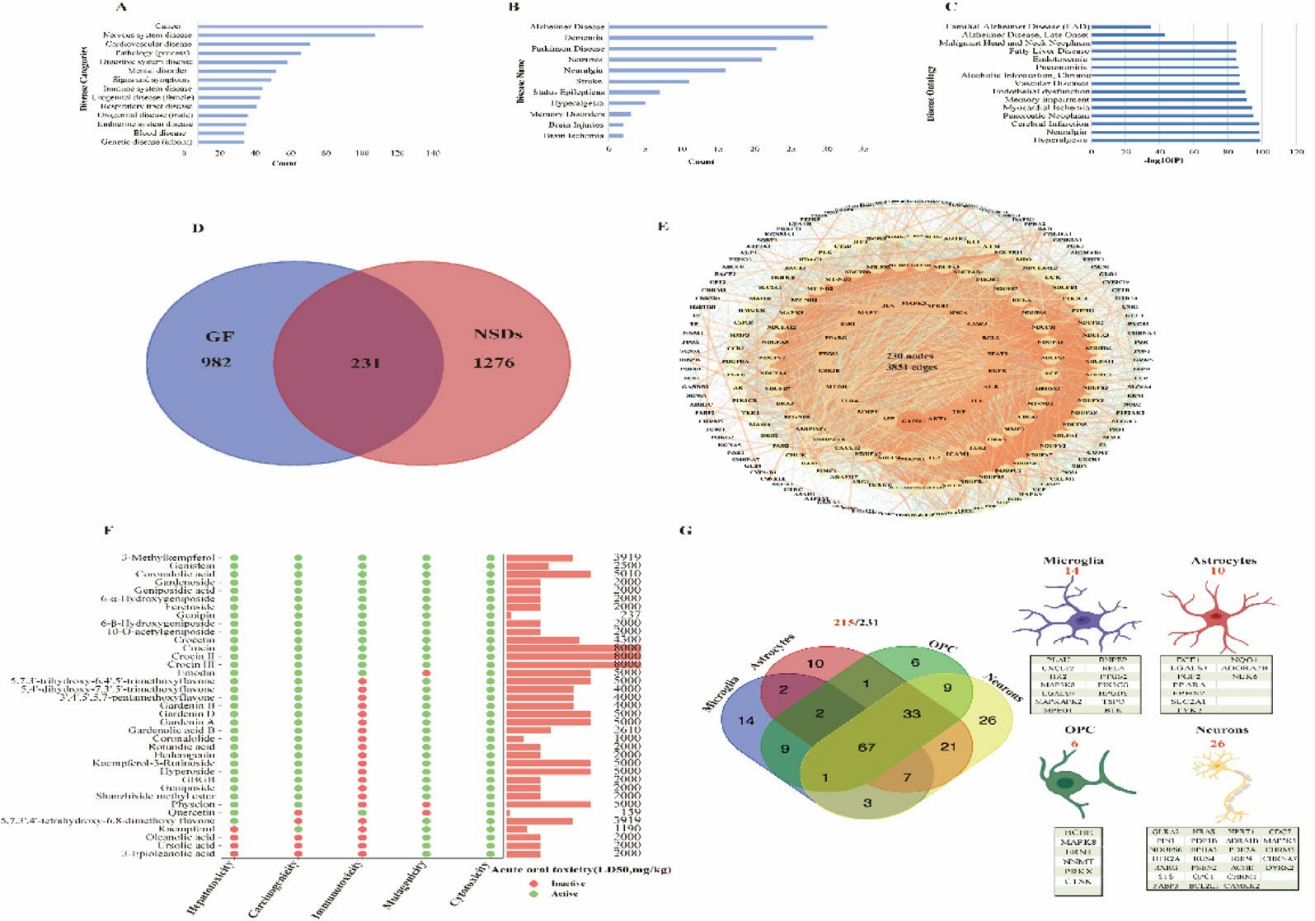
Screening and cellular localization of active compounds from Gardeniae Fructus with potential anti-NSDs activity. **A** Potential results analysis for disease types;** B** Potential results analysis for neurological diseases;** C** GF target disease ontology;** D** Venn diagram showing the overlap between NSDs targets and GF targets;** E** PPI network of 231 anti-NSDs targets of GF;** F** Pharmacological and toxicological parameters of major GF compounds;** G** Cellular classification of 231 targets of GF in treating NSDs

Literature and databases yielded 217 GF compounds, screened for activity using Swiss ADME. Some of these compounds met Lipinski’s Rule of Five (RO5), while others, although not fully complying with RO5, were reported in the literature to exhibit biological activity and were therefore included in the screening. Ultimately, 124 active compounds were identified (Table S1). Disease association profiling through Comparative Toxicogenomics Database (CTD) and Metascape revealed predominant neurological system involvement, particularly non-cancer neurodegenerative disorders (Alzheimer’s disease, dementia, Parkinson’s disease) as shown in Fig. 1A-B. Disease Ontology (DO) analysis (Fig. 1C) identified 1509 NSDs-related targets from GeneCard and OMIM databases, with 231 targets demonstrating neuroprotective relevance through intersection analysis (Fig. 1D). Protein-protein interaction (PPI) network construction generated a robust interactome (230 nodes, 3851 edges: Fig. 1E). Reverse pharmacophore screening coupled these 231 targets with the 124 bioactive compounds, yielding 37 candidates. ProTox-II toxicity filtering subsequently approved 33 non-toxic candidates for downstream analysis (Fig. 1F). To further clarify the core cellular targets of GF in the intervention of NSDs, this study imported 231 candidate targets into the AlzData database (v4.0), a multi-omics database for Alzheimer’s disease based on single-cell transcriptomics, for cell-type-specific enrichment analysis. The results showed that these targets were significantly enriched in neurons (26 targets), microglia (14 targets), astrocytes (10 targets), and oligodendrocyte precursor cells (OPCs, 6 targets) (Fig. 1G).

### Differences in the content of active compounds before and after processing of gardenia

**Fig. 2:**
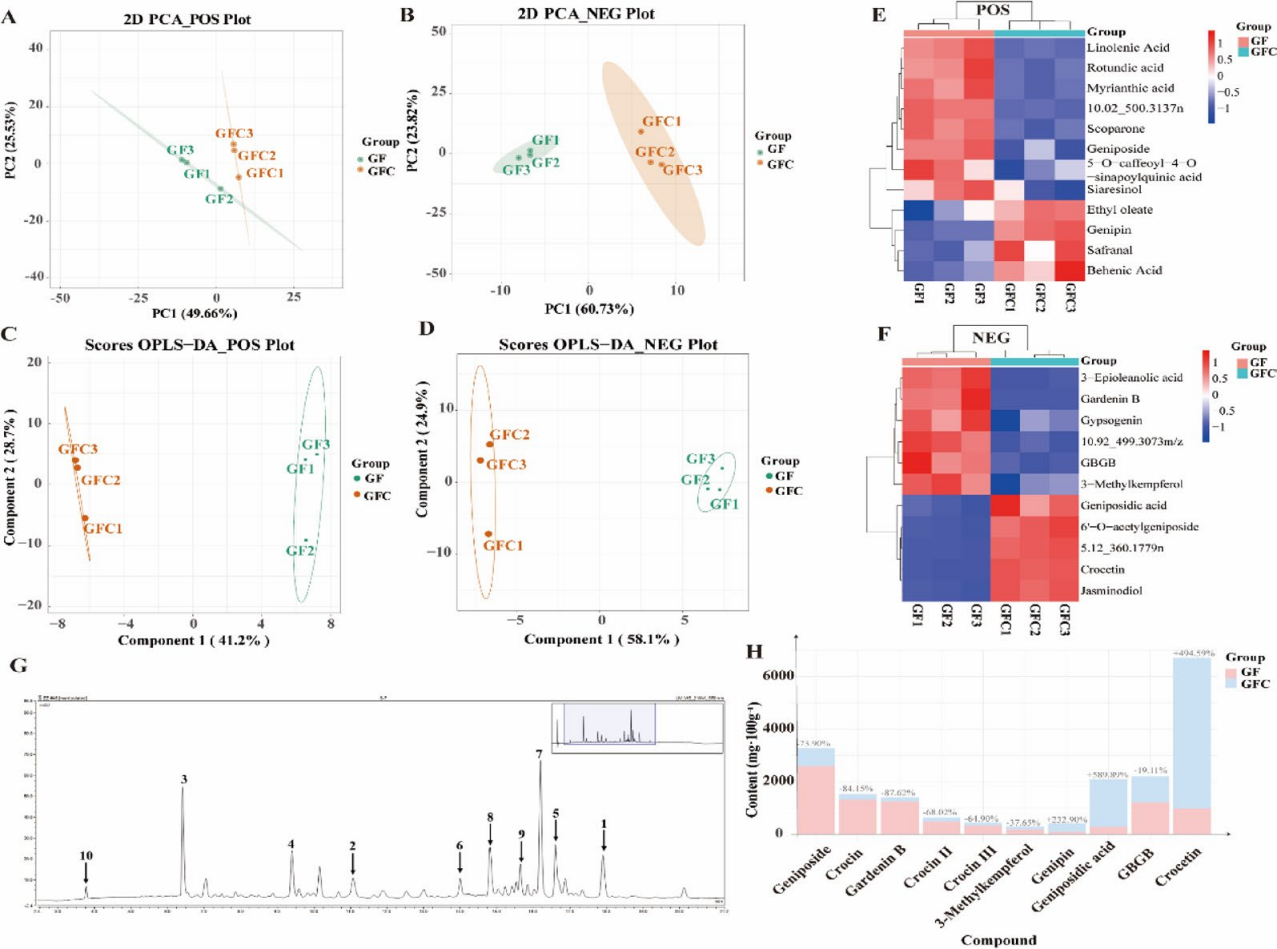
Chemical differences between GF and GFC revealed by UPLC-Q-TOF-MS/MS and quantification of key compounds.** A** PCA analysis of samples in positive ion mode;** B** OPLS-DA analysis in positive ion mode;** C** Heatmap of differential metabolites in positive ion mode;** D** PCA analysis of samples in negative ion mode;** E** OPLS-DA analysis in negative ion mode;** F** Heatmap of differential metabolites in negative ion mode;** G** UPLC-UV detection of GF at 370 nm;** H** Quantitative analysis of 10 differential compounds in GF and GFC extracts (mg·100 g⁻¹)

**Fig. 3:**
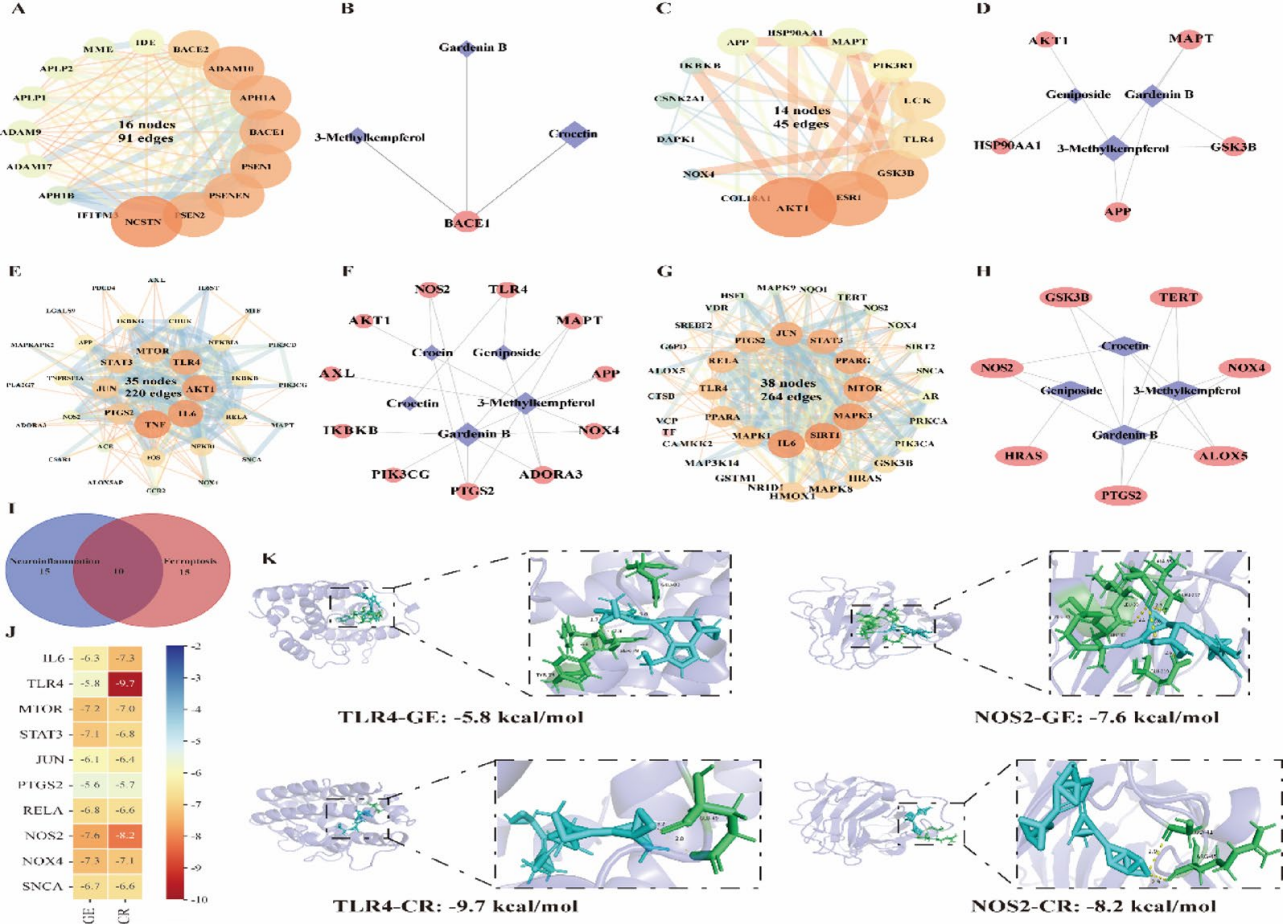
Network pharmacology and molecular docking revealing geniposide and crocetin as key neuroprotective compounds targeting neuroinflammation and ferroptosis.** A** PPI analysis of GF targets related to Aβ production and degradation;** B** GF-NSDs (Aβ)-target network;** C** PPI analysis of GF targets related to Tau protein phosphorylation;** D** GF-NSDs (Tau)-target network;** E** PPI analysis of GF targets related to neuroinflammation;** F** GF-NSDs (neuroinflammation)-target network;** G** PPI analysis of GF targets related to ferroptosis;** H** GF-NSDs (ferroptosis)-target network;** I** Venn diagram of the top 25 targets associated with neuroinflammation and ferroptosis;** J** Molecular docking heatmap of the overlapping targets between neuroinflammation and ferroptosis with GE and CR;** K** 3D visualization of molecular docking interactions between TLR4, NOS2, and GE and CR. **Note**: In the following figures, GE represents geniposide and CR represents crocetin. GE and CR are used to label these compounds consistently in subsequent figures

Using the supercritical CO_2_ extraction method, the extraction yield of GF was 14.91%, while that of GFC was reaching 19.73%. UPLC-Q-TOF-MS/MS was employed to comparatively analyze the phytochemical profiles of GF and GFC extracts (Fig. S1). To thoroughly examine phytochemical differences among sample groups, this work employed both unsupervised principal component analysis (PCA) and supervised orthogonal partial least squares-discriminant analysis (OPLS-DA) for multivariate data mining. As shown in Fig. 2A, the PCA model in positive ion mode showed a clear distinction between the GF and GFC data, with cumulative contribution rates of 75.19% (PC1: 49.66%, PC2: 25.53%). Enhanced discriminatory power emerged in negative ion mode, where cumulative variance explained increased to 84.55% (PC1: 60.73%, PC2: 23.82%), yielding more pronounced inter-group differentiation (Fig. 2B). OPLS-DA score plots delineated distinct spatial segregation between GF and GFC samples across ionization modes. In positive ion mode, the first predictive component explained 41.2% of X-variance, increasing to 58.1% in negative ion mode, confirming model efficacy in group discrimination (Figs. 3C-D). Variable importance in projection (VIP) screening (threshold > 1.0) identified 12 and 11 significantly differential metabolites (*p* < 0.05) in positive and negative modes respectively (Supplementary Tables S2- S3), yielding 23 potential biomarkers. Hierarchical clustering analysis (Fig. 3E-F) visualized pronounced concentration gradients of these biomarkers across groups. Intersection with prior network pharmacology findings (Fig. 1F) revealed 10 neuroprotection-associated compounds: Geniposide, Crocin, Gardenin B, Crocin II, Crocin III, 3-Methylkaempferol, Genipin, Geniposidic acid, GBGB, Crocetin.

Quantitative analysis based on UPLC-DAD demonstrated that the methodological validation parameters for the ten key compounds met international standards. The calibration curves exhibited a linear range of 0.20–60 ng/mL, with determination coefficients (R²) exceeding 0.9995, indicating excellent sensitivity and reliability of the method (Table S4). As shown in Fig. 2G and Table S4, compared to GF, the levels of Geniposide (−73.90%), Crocin (−84.15%), Gardenin B (−87.62%), Crocin II (−68.02%), Crocin III (-64.90%), 3-Methylkaempferol (−37.65%), GBGB (−19.11%), were significantly reduced in GFC (*p* < 0.01). In contrast, the levels of Genipin (+ 232.90%), Geniposidic acid (+ 589.89%), and Crocetin (+ 494.59%) were significantly increased (*p* < 0.05).

### Identification of bioactive compounds targeting distinct NSDs pathological mechanisms

AD, the leading neurodevelopmental disorder, features pathological Aβ aggregation (Tikhonova et al. 2021). A protein-protein interaction (PPI) network with 16 nodes and 91 edges was created after a thorough examination of molecular targets linked to Aβ generation and degradation pathways identified 17 crucial targets (Fig. 3A). Subsequent network pharmacology analysis of the Differential components (DC)-NSDs (Aβ)-target network identified three bioactive compounds demonstrating anti-Aβ deposition potential: 3-Methylkaempferol, Gardenin B, and Crocetin (Fig. 3B). The pathological cascade of AD further involves Tau protein hyperphosphorylation, which promotes neurofibrillary tangle formation, disrupts neuronal cytoarchitecture, and exacerbates neurodegenerative progression (Grill and Cummings 2010). Through comprehensive target screening, we identified 14 targets associated with Tau phosphorylation, including 14 nodes and 45 edges (Fig. 3C). Network analysis of the DC-NSDs (Tau)-target system (Fig. 3D) highlighted three compounds with anti-phosphorylation activity: 3-Methylkaempferol, Geniposide, and Gardenin B. Neuroinflammation constitutes a pivotal driver of NSDs progression (Hammond et al. 2019). Through integrated bioinformatics analysis combining PubMed mining and Gene Ontology term screening (GO:0006954, GO:0002675, GO:0050729, GO:0001774), we identified 38 neuroinflammation-associated GF targets and constructed a protein interaction network consisting of 35 nodes and 220 edges. (Fig. 3E). Subsequent network pharmacology evaluation of the DC-NSDs (neuroinflammation)-target axis (Fig. 3F) revealed five potential anti-neuroinflammatory compounds: Geniposide, 3-Methylkaempferol, Crocin, Gardenin B, and Crocetin. Ferroptosis, an iron-regulated programmed cell death mechanism, has emerged as a critical contributor to NSDs pathogenesis (Bao et al. 2021). Utilizing the FerrDb ferroptosis database, a total of 38 genes linked to the process of ferroptosis were pinpointed. Furthermore, an intricate protein-protein interaction network was assembled, featuring 38 interconnected nodes and 264 connecting lines (Fig. 3G). Systematic analysis of the DC-AD (ferroptosis)-target interactome (Fig. 3H) identified four ferroptosis-modulating compounds: 3-Methylkaempferol, Gardenin B, Geniposide, and Crocetin.

Enrichment analysis revealed that compounds associated with neuroinflammation and ferroptosis exhibited the most significant enrichment effects. Notably, Geniposide and Crocetin not only showed relatively high content among the original components of GF but also demonstrated distinct dynamic changes during the processing of the herb. Therefore, we selected these two active compounds as the primary focus of our investigation. Further analysis identified significant overlap in key targets between the two pathological mechanisms of neuroinflammation and ferroptosis, suggesting that these pathways may represent the core pharmacological routes through which the active components of GF exert their effects. Based on this finding, we performed an intersection analysis of the core targets of both pathological mechanisms, ultimately identifying 10 shared core targets (Fig. 3I). We used molecular docking techniques to examine Geniposide and Crocetin against each of the ten main targets in order to better understand the interactions between the active molecules and these core targets. The docking results revealed particularly strong binding affinities with TLR4 and NOS2, indicating that these two target proteins may exhibit higher binding potential with the active compounds (Fig. 3J-K).

### Geniposide and Crocetin attenuate LPS-Induced inflammation in BV2 cells

Analysis of Fig. 1G revealed that Gardenia GF-related NSDs targets were predominantly enriched in microglia and neuronal cells. To further validate the potential mechanisms of action of Geniposide and Crocetin, we conducted subsequent functional verification experiments using BV2 microglial cell lines and HT22 neuronal cell models.

**Fig. 4:**
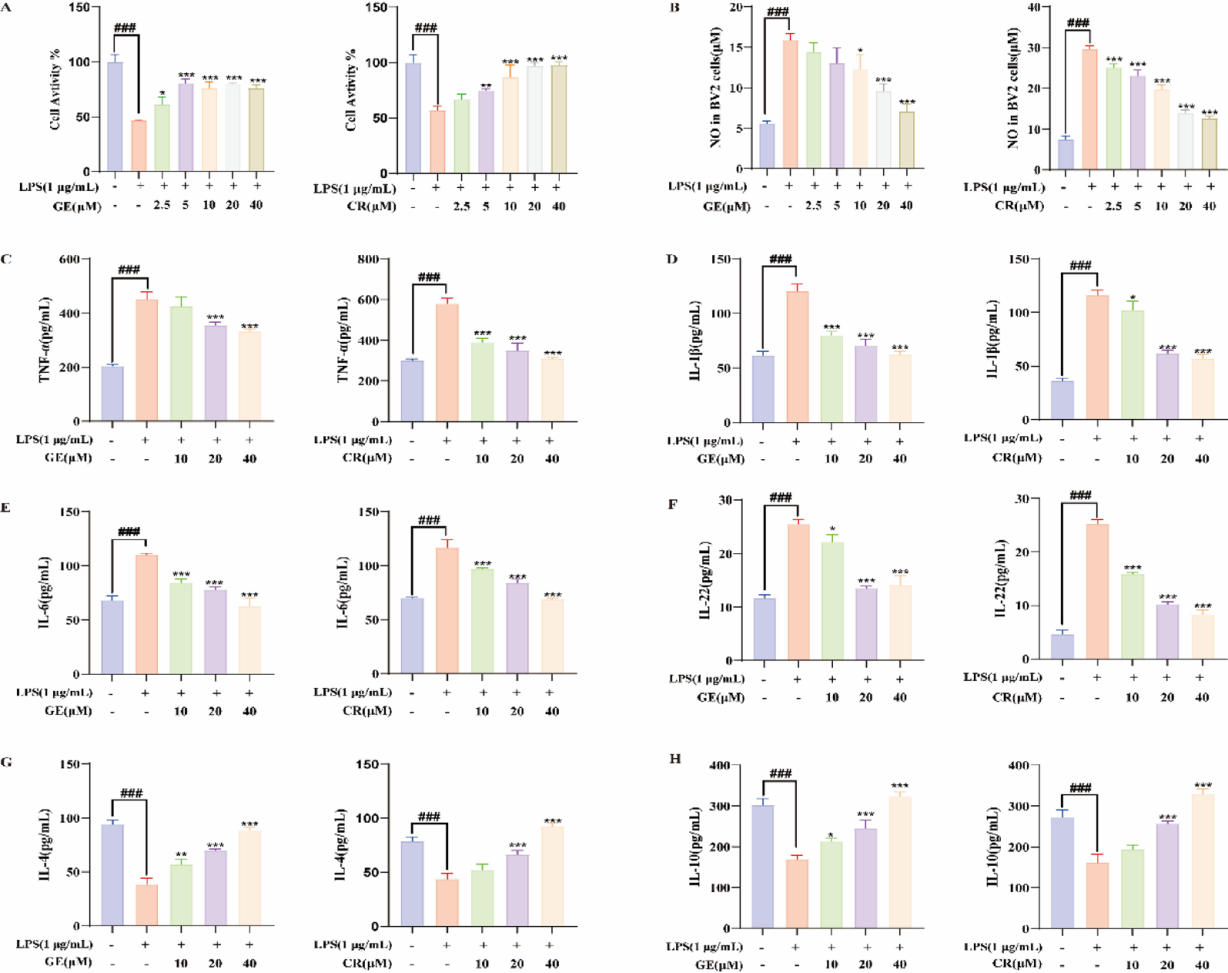
Effects of geniposide and crocetin on LPS-induced inflammatory responses in BV2 microglial cells. **A** Cell viability assay; **B** NO content measurement; pro-inflammatory cytokine levels of TNF-α **C**, IL-1β (D), IL-6 **E**, and IL-22 **F**; anti-inflammatory cytokine levels of IL-4 **G** and IL-10 **H** (*p < 0.05, **p < 0.01, ***p < 0.001 vs. LPS; #*p* < 0.05, ##*p* < 0.01, ###*p* < 0.001 vs. control)

In preliminary experiments, Gardenin B (2) and Genipin (6) exhibited slight toxicity to BV2 cells (Fig. S2A), whereas both GF and GFC extracts (0–200 µg/mL) and other compound treatments (40 µM) had no effect on the proliferation of LPS-stimulated BV2 cells (*p* < 0.001, Figs. 4A, S2B–C). In LPS-induced NO production assays, both GF and GFC extracts (200 µM) exhibited dose-dependent reductions in NO levels compared to the control group (*p* < 0.001), decreasing levels by 7–11.32%, respectively (Fig.S2 D). Geniposide (20 µM) and crocetin (20 µM) reduced NO levels by 22.5–33.1%, respectively (*p* < 0.001; Fig. 4B). Regarding pro-inflammatory cytokines, both GF and GFC extracts (0–200 µg/mL) dose-dependently inhibited the secretion of TNF-α (Fig. S2E), IL-1β (Fig. S2F), IL-6 (Fig. S2G), and IL-22 (Fig. S2H), while significantly enhancing the secretion of anti-inflammatory cytokines IL-4 (Fig. S2I) and IL-10 (Fig. S2J). In comparison, geniposide (20 µM) significantly suppressed the secretion of TNF-α (Fig. 4C), IL-1β (Fig. 4D), IL-6 (Fig. 4E), and IL-22 (Fig. 4F) by 21.0%, 29.2%, 41.6%, and 47.0%, respectively, compared to controls (*p* < 0.001). Crocetin (20 µM) produced greater reductions of 39.5%, 27.9%, 47.5%, and 59.7%, respectively. Both compounds significantly promoted the secretion of anti-inflammatory cytokines (*p* < 0.001). Specifically, geniposide increased IL-4 (Fig. 4G) and IL-10 (Fig. 4H) levels by 80.3– 45.6%, respectively, while crocetin elevated them by 52.0–57.9%, compared to the control.

### Geniposide and Crocetin protect HT22 cells from Erastin-Induced ferroptosis

**Fig. 5:**
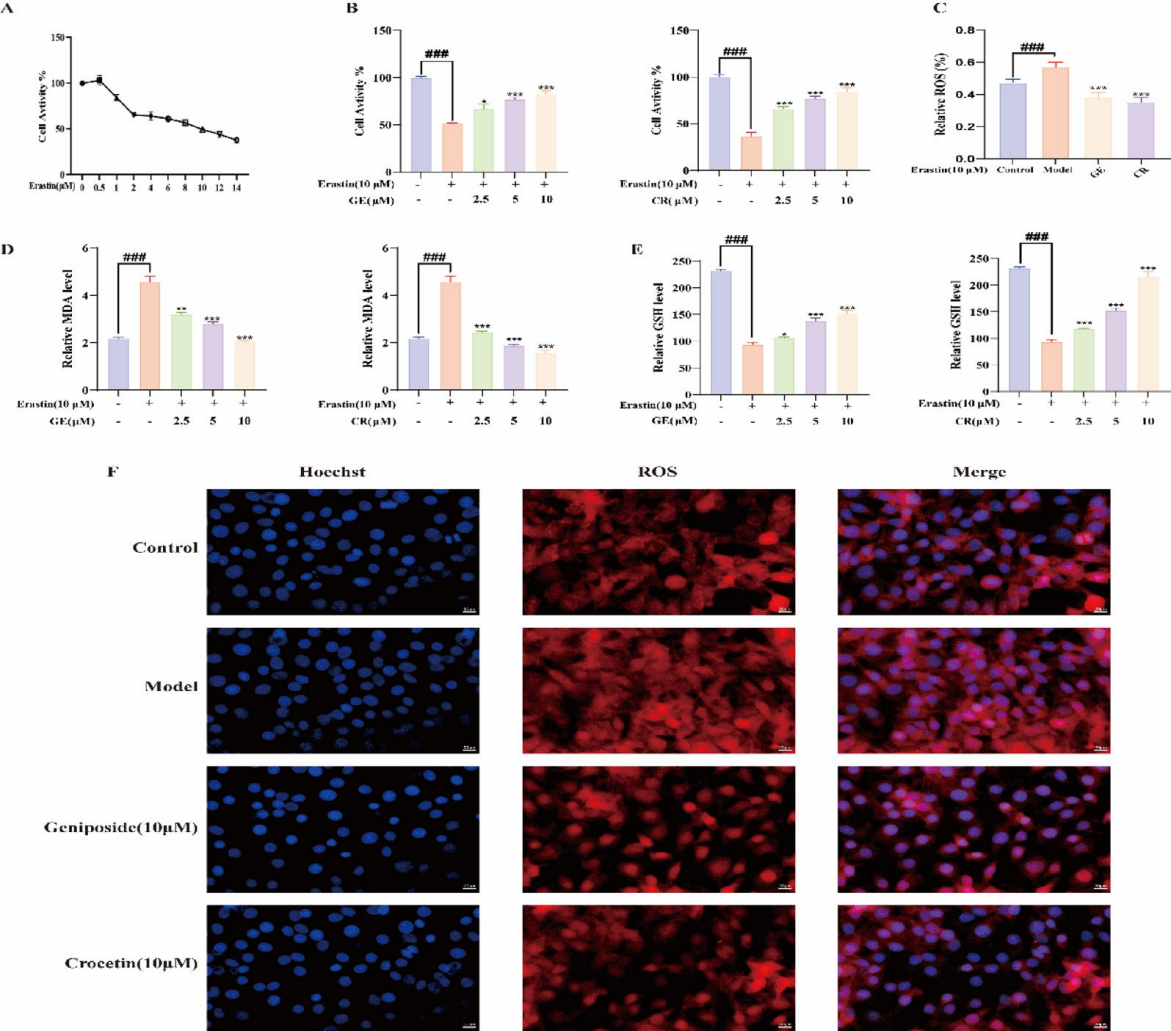
Protective effects of geniposide and crocetin against erastin-induced ferroptosis in HT22 hippocampal neurons. **A** Selection of optimal concentration for Erastin-induced HT22 cell injury; **B** Cell viability assay; **C** ROS level detection; **D** MDA content detection; **E** GSH content detection; **F** Immunofluorescence detection of ROS levels. (*p < 0.05, **p < 0.01, ***p < 0.001 vs. Erastin; #*p* < 0.05, ##*p* < 0.01, ###*p* < 0.001 vs. control)

In preliminary experiments, we found that the compounds (0–10 µM) were non-toxic to HT22 cells, and both GF and GFC extracts (0–75 µg/mL) as well as compound treatments (10 µM) had no effect on Erastin-induced HT22 cell proliferation (*p* < 0.001, Fig. S2L–M). The optimal induction concentration was 10 µM Erastin, which decreased cell viability in HT22 cells to 49.3% as compared to the blank group (Fig. 5A). GF and GFC extracts enhanced cell viability in the Erastin-induced HT22 cell culture in a dose-dependent manner (*p* < 0.001, Fig. S2K). Furthermore, geniposide (10 µM) and crocetin (10 µM) enhanced cell viability by 59.1% and 86.91%, respectively, in comparison to the control group (*p* < 0.001, Fig. 5B).

Lipid peroxidation and its metabolites also showed significant changes. Erastin treatment threw MDA levels way up while simultaneously tanking GSH levels when compared to the control group. In the Erastin-induced ferroptosis model, both GF and GFC crude extracts significantly inhibited MDA levels (Fig. S2N) and restored GSH levels (*p* < 0.001, Fig. S2O). But get this: both geniposide and crocetin really put the brakes on those elevated MDA levels in Erastin-stressed HT22 cells, and they did so more effectively as the dosage increased (*p* < 0.001). Specifically, Geniposide (10 µM) reduced MDA levels by 54.99%, and Crocetin (10 µM) by 65.92% compared to the control group (Fig. 5D). Meanwhile, both compounds significantly increased GSH levels (*p* < 0.001), with Geniposide (10 µM) increasing by 62.63% and Crocetin (10 µM) by 131.89% (Fig. 5E). Laser confocal microscopy results showed that ROS levels were significantly increased in the Erastin group compared to the control group (Fig. 5F). Geniposide (10 µM) decreased ROS levels by 32.98%, while Crocetin (10 µM) decreased them by 38.54% (Fig. 5C).

### Neuroprotection by Geniposide and Crocetin via TLR4/NF-κB suppression and Nrf2 activation

**Fig. 6:**
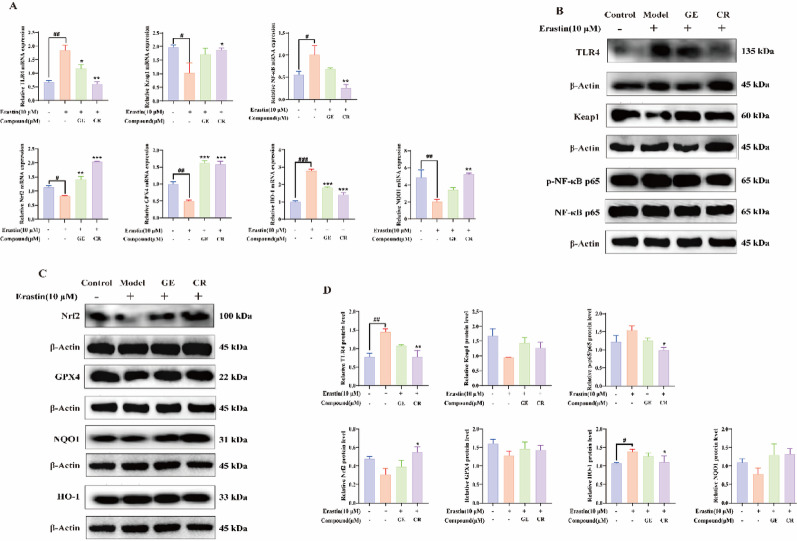
Effects of GE and CR on TLR4/NF-κB and Nrf2-antioxidant pathways in erastin-treated HT22 cells. **A** qRT-PCR analysis of related genes; **B** Protein expression of TLR4, Keap1, and NF-κB; **C** Protein expression of GPX4, NQO1, HO-1, and Nrf2; **D** Western blot analysis of protein levels. (*p < 0.05, **p < 0.01, ***p < 0.001 vs. Erastin; #*p* < 0.05, ##*p* < 0.01, ###*p* < 0.001 vs. control)

Ferroptosis disrupted the antioxidant system, as evidenced by the significantly downregulated mRNA levels of Nrf2, Keap1, GPX4, and NQO1 (*p* < 0.001) and significantly upregulated mRNA expression of TLR4, NF-κB, and HO-1 (*p* < 0.001) in Erastin-induced HT22 cells compared to the control group. Following treatment with Geniposide and Crocetin, the expression of TLR4, NF-κB, and HO-1 was significantly reduced, with Crocetin showing a stronger inhibitory effect. Conversely, Nrf2, Keap1, GPX4, and NQO1 expression levels were significantly upregulated, with Crocetin exhibiting a more pronounced enhancement effect (Fig. 6A).

Western blotting verified that Erastin increased TLR4, NF-κB, and HO-1 but decreased Nrf2, Keap1, GPX4, and NQO1 protein expression. Treatment with Geniposide and Crocetin reversed these trends, with Crocetin exerting a stronger downregulatory effect on TLR4, NF-κB, and HO-1, as well as a more pronounced upregulation of Nrf2, Keap1, GPX4, and NQO1 (Fig. 6B, C, D). These findings suggest that Geniposide and Crocetin alleviate ferroptosis by inhibiting the TLR4/NF-κB pathway and activating the Nrf2-Keap1 axis, with Crocetin demonstrating superior neuroprotective potential.

## Discussion

This study aims to systematically investigate the chemical transformations of GF during processing and explore the pharmacodynamic significance of its key active components in pathological mechanisms potentially related to neuroinflammation and ferroptosis. By comparing GF extract with its processed counterpart (GFC), preliminary analysis identified 23 differential metabolites, among which geniposide and crocetin appeared as potential core components with possible therapeutic relevance. Our findings provide initial evidence at the molecular level regarding the dynamic changes in chemical constituents during gardenia processing and their potential correlation with biological activity, offering a perspective worth further exploration for validating traditional Chinese medicine processing techniques.

Although the Chinese Pharmacopoeia currently designates geniposide as the sole quality control marker for GF and GFC, UPLC-DAD quantitative analysis revealed that high-temperature processing significantly reduced water-soluble glycosides such as geniposide, crocin, and crocin II/III, while corresponding deglycosylated products (genipin, crocetin) and oxidation/hydrolysis products (geniposidic acid) increased markedly. To further elucidate the chemical basis underlying these differences, our quantitative analyses revealed typical thermal transformation patterns during gardenia processing. Multiple glycosidic constituents—including geniposide, crocin series, and related derivativeswere markedly reduced after heating, consistent with the intrinsic thermal instability of glycosides that undergo glycosidic bond cleavage, deglycosylation, and subsequent oxidative fragmentation under high-temperature conditions (Dong et al. 2022). Concurrently, substantial increases in genipin and geniposidic acid indicate that geniposide underwent heat-induced deglycosylation, ring-opening, and structural rearrangement to form downstream stable products (Xu et al. 2021). Likewise, the sharp decrease in crocin accompanied by a significant accumulation of its aglycone crocetin provides strong evidence that carotenoid glycosides are prone to thermal deglycosylation, with the resulting aglycones exhibiting greater stability (Narra et al. 2024). These findings collectively outline a characteristic “glycoside loss–aglycone enrichment” pattern during processing, offering a structural and experimental basis for the elevated crocetin levels and enhanced bioactivities observed in GFC. These observations highlight the potential need to consider additional bioactive compounds (e.g., crocetin) in quality assessment protocols. This possibility is of particular interest, as the elevated crocetin content in GFC might partially account for its prolonged clinical use in TCM. By more comprehensively accounting for changes in key components during processing, the pharmacological characteristics of processed gardenia can be better reflected, thereby enhancing the scientific rigor and practicality of quality standards for traditional Chinese medicine.

Importantly, these chemical transformations may also imply shifts in pharmacological effects. Based on their chemical structures, genipin and crocetin, as aglycones derived from geniposide and crocin, possess reduced polarity and are predicted to have higher lipophilicity and improved membrane permeability. This structural feature may contribute to enhanced bioavailability and therapeutic potential in certain disease models. Conversely, the reductions in geniposide and crocin, which are highly polar glycosides, could decrease water-soluble-related pharmacological effects such as heat-clearing or antioxidant activities. Thus, traditional processing “reshapes” GF’s chemical composition, partially altering its pharmacological spectrum. From a safety and quality perspective, high-temperature processing may have dual effects: stabilizing unstable or bitter compounds, while potentially generating trace thermal degradation or browning products. Although such byproducts were not detected in this study, future high-resolution mass spectrometry analyses are warranted.

Mechanistically, neuroinflammation and ferroptosis have emerged as two core pathological processes in GF-related diseases, with their key molecular targets showing significant overlap. This suggests they may collectively constitute the central pharmacological pathways of GF’s active components. Literature evidence indicates that TLR4 signaling pathway activation triggers the release of pro-inflammatory factors (NO, ROS, etc.) via the NF-κB pathway. These inflammatory mediators form a pathological cascade with neuronal ferroptosis, synergistically driving the progression of NSDs (Deleidi and Gasser 2013; Ward et al. 2022; You et al. 2017). NO is a key and widely recognized marker of neuroinflammation in LPS-stimulated BV2 microglial cells (Aktan 2004; Yuste et al. 2015). GF extract, GFC extract, geniposide, and crocetin exhibited inhibition of NO and pro-inflammatory cytokines (such as TNF-α, IL-1β and IL-6), while also promoting anti-inflammatory cytokines (such as IL-4 and IL-10) in LPS-stimulated BV2 cells. Notably, in an Erastin-induced HT22 ferroptosis model, crocetin demonstrated greater inhibition of the TLR4/NF-κB pathway, a trend supported by molecular docking analysis indicating higher TLR4 binding affinity for crocetin. From a molecular mechanistic perspective, TLR4/NF-κB pathway activation induces ROS generation (Cheng et al. 2021; Yang et al. 2022), and ROS, as critical secondary messengers, can activate the Nrf2 signaling system. Under basal conditions, Nrf2 undergoes rapid degradation via Keap1 binding, upon ROS stimulation, Nrf2 dissociates from Keap1 and translocates to the nucleus (Dodson et al. 2019; Stockwell 2022). Our data further suggest that crocetin could upregulate antioxidant enzymes GPX4 and NQO1 via Nrf2 activation, implying a dual role in modulating neuroinflammation and ferroptosis. However, these findings require validation in more complex biological systems.

Additionally, this study attempted to link gardenia’s components to neuroinflammation and ferroptosis through pathological mechanism specific network pharmacology, providing preliminary insights for ethnopharmacological research. While this approach offers methodological innovation, its predictive value needs confirmation through functional studies. Several limitations should be noted. Although* in vitro* models provide mechanistic clues, the complexity of neurodegenerative diseases necessitates in vivo validation. While the TLR4/NF-κB/Nrf2 axis appears central, other pathways might contribute and warrant investigation.

In conclusion, this work offers preliminary support for traditional gardenia processing methods by integrating chemical analysis with mechanistic studies. Our results align with the hypothesis that processing alters compositional profiles, potentially enhancing therapeutic effects. This framework could serve as a starting point for studying other herbal processing techniques, though broader applicability requires further exploration.

## Supplementary Information

Below is the link to the electronic supplementary material.

Supplementary Material 1

## Data Availability

The datasets utilized and examined in the present study can be obtained upon a reasonable request from the corresponding author.

## Abbreviations

GO: Gene ontology
KEGG: Kyoto Encyclopedia of Genes and Genomes
OB: Oral bioavailability
DL: Drug-likeness
PDB: Protein Data Bank
PVDF: Polyvinylidene fluoride
DMEM: Dulbecco’s modified Eagle’s medium
RT-qPCR: Reverse transcription-quantitative polymerase chain reaction
PPI: Protein-protein interaction
GSH: Glutathione
MDA: Malondialdehyde
ROS: Reactive Oxygen Species
TLR4: Toll-Like Receptor 4
NF-κB: Nuclear Factor kappa-light-chain-enhancer of activated B cells
Nrf2: Nuclear factor erythroid 2-related factor 2
Keap1: Kelch-like ECH-associated protein 1
GPX4: Glutathione Peroxidase 4
NQO1: NAD(P)H Quinone Dehydrogenase 1
HO-1: Heme Oxygenase 1
