# Supplementary material for: Processing-induced changes in neuroprotective components and mechanisms of gardeniae fructus: integrating UPLC-Q-TOF-MS/MS, network pharmacology, and in vitro analysis
Source: Bioresour Bioprocess. 2026 Jan 27;13(1):10. doi: 10.1186/s40643-025-01005-0 (PMC12835472; doi:10.1186/s40643-025-01005-0)
Supplement: Supplementary file 1 — Supplementary Material 1 [file 40643_2025_1005_MOESM1_ESM.docx]

**Supplementary Materials**

**Processing-Induced Changes in Neuroprotective Components and Mechanisms of *Gardeniae Fructus*: Integrating UPLC-Q-TOF-MS/MS, Network Pharmacology, and In Vitro Analysis**

Le Sun ^a#^, Ziyu Hou ^a#^, Wenjie Wang ^a^, Peiling Wu ^a^, Pei Ma ^a^, Jiali Huang ^a^, Leyang Fan ^a^, Lijia Xu ^a*^, Haibo Liu ^a*^, Peigen Xiao ^a^

^a^ *Institute of Medicinal Plant Development, Chinese Academy of Medical Sciences and Peking Union Medical College, Beijing, China*

*Correspondence:

**Lijia Xu**

Email: ljxu@implad.ac.cn

Address: Institute of Medicinal Plant Development (IMPLAD), Chinese Academy of Medical Sciences & Peking Union Medical College

No. 151 Malianwa North Road, Haidian District, Beijing 100193, P.R. China

**Haibo Liu**

Email: hbliu@implad.ac.cn

Address: Institute of Medicinal Plant Development (IMPLAD), Chinese Academy of Medical Sciences & Peking Union Medical College

No. 151 Malianwa North Road, Haidian District, Beijing 100193, P.R. China

Author Contributions:

^#^Le Sun and Ziyu Hou contributed equally to this work.


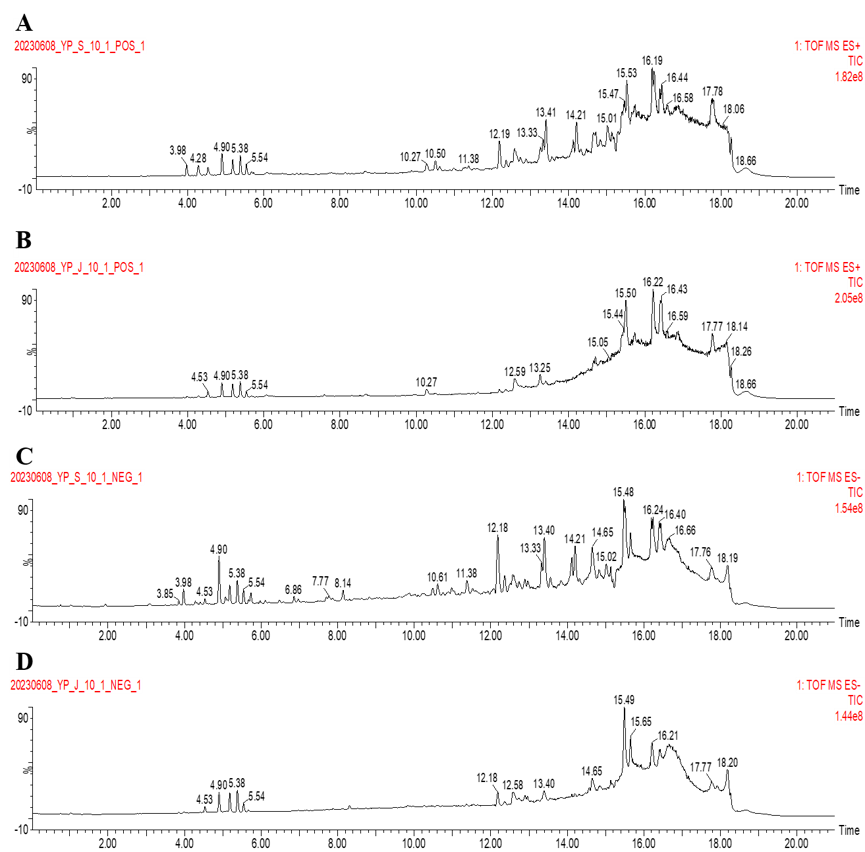
**Figure S1.** Liquid chromatograms for positive and negative ion detection: (A) Positive ion chromatogram of GF, (B) Positive ion chromatogram of GFC, (C) Negative ion chromatogram of GF, (D) Negative ion chromatogram of GFC.

**
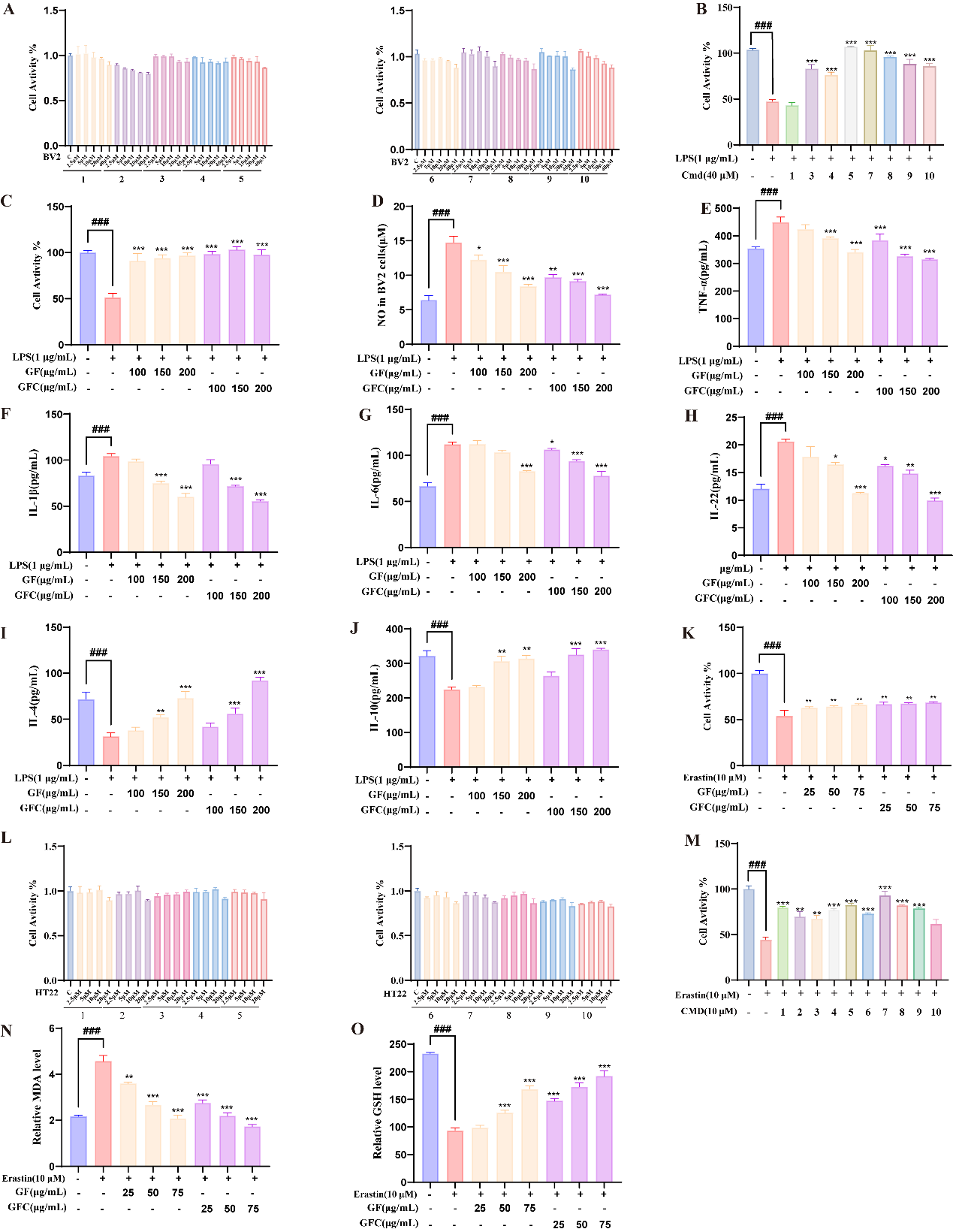
Figure S2.** Activity of GF and GFC extracts and compounds in BV2 and HT22 cell models. (A) Effects of 10 compounds on BV2 cell proliferation (CCK-8 cytotoxicity); (B) Effects of 8 compounds on the viability of LPS-induced BV2 cells; (C) Effects of GF and GFC extracts on the viability of LPS-induced BV2 cells; (D) Effects of GF and GFC extracts on nitric oxide (NO) levels in LPS-induced BV2 cells; (E–J) Effects of GF and GFC extracts on the secretion of inflammatory cytokines (TNF-α, IL-1β, IL-6, IL-22, IL-4, IL-10) in LPS-induced BV2 cells; (K) Effects of GF and GFC extracts on the viability of Erastin-induced HT22 cells; (L) Effects of 10 compounds on HT22 cell proliferation (CCK-8 cytotoxicity); (M) Effects of 10 compounds on the viability of Erastin-induced HT22 cells; (N) Effects of GF and GFC extracts on MDA levels in Erastin-induced HT22 cells; (O) Effects of GF and GFC extracts on GSH levels in Erastin-induced HT22 cells.

Note: Compound numbers 1–10 shown in the figure correspond to the compound IDs listed in Table S4. Data shown as Means ± SD (n=3), compared with the CON group, ^#^*p* < 0.05, ^##^*p* < 0.01, ^###^*p* < 0.001; compared with the MOD group, ^*^*p* ≤ 0.05, ^**^*p* < 0.01, ^***^*p* < 0.001.

**Table S1:** Screening of 124 Active Compounds Complying with Lipinski’s Rule of Five (RO5)

| **No.** | **Compound** | **MW** | **LogP** | **Hdon** | **Hacc** | **Rbon** |
| --- | --- | --- | --- | --- | --- | --- |
| 1 | CID_5281232 | 328.4 | 3.67 | 2 | 4 | 8 |
| 2 | CID_11869658 | 456.7 | 3.67 | 2 | 3 | 1 |
| 3 | CID_10212 | 270.28 | 3.05 | 0 | 4 | 3 |
| 4 | CID_62331 | 352.39 | 4.14 | 1 | 5 | 4 |
| 5 | CID_5280343 | 302.24 | 1.63 | 5 | 7 | 1 |
| 6 | CID_5280863 | 286.24 | 1.7 | 4 | 6 | 1 |
| 7 | CID_68081 | 270.28 | 3.07 | 0 | 4 | 3 |
| 8 | CID_5280862 | 300.26 | 2.11 | 3 | 6 | 2 |
| 9 | CID_107848 | 388.37 | 2.48 | 5 | 10 | 6 |
| 10 | CID_64945 | 456.7 | 3.95 | 2 | 3 | 1 |
| 11 | CID_12315075 | 488.7 | 3.45 | 4 | 5 | 2 |
| 12 | CID_503737 | 418.39 | 1.46 | 5 | 9 | 4 |
| 13 | CID_19009 | 352.4 | 0.1 | 0 | 4 | 4 |
| 14 | CID_72323 | 338.38 | -0.15 | 1 | 4 | 3 |
| 15 | CID_10807517 | 336.36 | 0 | 0 | 4 | 2 |
| 16 | CID_10114 | 470.68 | 3.46 | 2 | 4 | 1 |
| 17 | CID_1183 | 152.15 | 1.57 | 1 | 3 | 2 |
| 18 | CID_689043 | 180.16 | 0.97 | 3 | 4 | 2 |
| 19 | CID_72 | 154.12 | 0.66 | 3 | 4 | 1 |
| 20 | CID_92825 | 470.68 | 3.01 | 2 | 4 | 2 |
| 21 | CID_10494 | 456.7 | 3.94 | 2 | 3 | 1 |
| 22 | CID_73299 | 472.7 | 3.48 | 3 | 4 | 2 |
| 23 | CID_12315525 | 472.7 | 3.51 | 3 | 4 | 1 |
| **No.** | **Compound** | **MW** | **LogP** | **Hdon** | **Hacc** | **Rbon** |
| 24 | CID_8785 | 150.17 | 2.13 | 0 | 2 | 3 |
| 25 | CID_7150 | 136.15 | 1.98 | 0 | 2 | 2 |
| 26 | CID_5284507 | 222.37 | 3.64 | 1 | 1 | 7 |
| 27 | CID_11594 | 114.23 | 2.78 | 0 | 0 | 4 |
| 28 | CID_6184 | 100.16 | 1.77 | 0 | 1 | 4 |
| 29 | CID_7237 | 106.17 | 2.03 | 0 | 0 | 0 |
| 30 | CID_61041 | 150.22 | 2.13 | 0 | 1 | 1 |
| 31 | CID_6918391 | 204.35 | 3.37 | 0 | 0 | 3 |
| 32 | CID_6436348 | 218.33 | 2.96 | 0 | 1 | 0 |
| 33 | CID_18591 | 128.26 | 3.02 | 0 | 0 | 5 |
| 34 | CID_16664 | 128.26 | 3.02 | 0 | 0 | 5 |
| 35 | CID_16665 | 128.26 | 2.95 | 0 | 0 | 5 |
| 36 | CID_445070 | 222.37 | 2.56 | 1 | 1 | 7 |
| 37 | CID_102611 | 170.25 | 2.56 | 1 | 2 | 4 |
| 38 | CID_6549 | 154.25 | 2.7 | 1 | 1 | 4 |
| 39 | CID_17141 | 190.28 | 1.75 | 3 | 3 | 1 |
| 40 | CID_5283356 | 168.28 | 2.87 | 0 | 1 | 8 |
| 41 | CID_7362 | 96.08 | 1.03 | 0 | 2 | 1 |
| 42 | CID_6234 | 102.09 | 0.53 | 2 | 3 | 0 |
| 43 | CID_417575 | 100.12 | 1.2 | 1 | 2 | 2 |
| 44 | CID_8452 | 84.12 | 1.38 | 0 | 1 | 0 |
| 45 | CID_6054 | 122.16 | 1.7 | 1 | 1 | 2 |
| 46 | CID_6883 | 236.14 | 0.59 | 0 | 8 | 4 |
| 47 | CID_5281327 | 398.66 | 4.82 | 1 | 1 | 4 |
| **No.** | **Compound** | **MW** | **LogP** | **Hdon** | **Hacc** | **Rbon** |
| 48 | CID_5281328 | 412.69 | 4.97 | 1 | 1 | 5 |
| 49 | CID_261859 | 418.39 | 3.79 | 1 | 9 | 7 |
| 50 | CID_96539 | 358.34 | 3.57 | 1 | 7 | 5 |
| 51 | CID_3080750 | 374.34 | 3.07 | 2 | 8 | 5 |
| 52 | CID_13786166 | 204.18 | 1.99 | 0 | 4 | 3 |
| 53 | CID_10775442 | 223.23 | 1.92 | 2 | 4 | 3 |
| 54 | CID_5317764 | 354.35 | 2.56 | 4 | 6 | 3 |
| 55 | CID_76551288 | 330.37 | 1.94 | 4 | 7 | 4 |
| 56 | CID_21603216 | 360.36 | 1.22 | 5 | 9 | 4 |
| 57 | CID_98050833 | 330.37 | 1.77 | 4 | 7 | 4 |
| 58 | CID_102507170 | 346.37 | 2.1 | 5 | 8 | 4 |
| 59 | CID_24896698 | 184.23 | 2.12 | 2 | 3 | 2 |
| 60 | CID_102507168 | 346.37 | 1.62 | 5 | 8 | 5 |
| 61 | CID_102507169 | 328.36 | 1.7 | 4 | 7 | 4 |
| 62 | CID_21631030 | 330.37 | 2.18 | 4 | 7 | 4 |
| 63 | CID_10807517 | 346.37 | 1.69 | 5 | 8 | 4 |
| 64 | CID_15765124 | 212.2 | 1.43 | 3 | 5 | 4 |
| 65 | CID_8417 | 206.19 | 2.23 | 0 | 4 | 2 |
| 66 | CID_5280460 | 192.17 | 1.86 | 1 | 4 | 1 |
| 67 | CID_8742 | 174.15 | 0.51 | 4 | 5 | 1 |
| 68 | CID_6782 | 278.34 | 3.31 | 0 | 4 | 8 |
| 69 | CID_3026 | 278.34 | 2.97 | 0 | 4 | 10 |
| 70 | CID_64971 | 456.7 | 3.81 | 2 | 3 | 2 |
| 71 | CID_10742 | 198.17 | 1.54 | 2 | 5 | 3 |
| **No.** | **Compound** | **MW** | **LogP** | **Hdon** | **Hacc** | **Rbon** |
| 72 | CID_8468 | 168.15 | 1.4 | 2 | 4 | 2 |
| 73 | CID_8655 | 182.17 | 1.66 | 1 | 4 | 3 |
| 74 | CID_69505 | 184.19 | 1.95 | 1 | 4 | 3 |
| 75 | CID_31244 | 136.15 | 1.68 | 0 | 2 | 2 |
| 76 | CID_442424 | 226.23 | 1.94 | 2 | 5 | 3 |
| 77 | CID_78066 | 166.17 | 1.84 | 1 | 3 | 3 |
| 78 | CID_11631807 | 358.34 | 0.99 | 5 | 9 | 4 |
| 79 | CID_133626 | 390.38 | 1.83 | 5 | 10 | 5 |
| 80 | CID_11105753 | 168.23 | 1.91 | 1 | 2 | 1 |
| 81 | CID_102248364 | 448.64 | 3.91 | 3 | 5 | 0 |
| 82 | CID_10611205 | 258.27 | 2.26 | 2 | 6 | 7 |
| 83 | CID_6479499 | 482.65 | 3.64 | 1 | 5 | 8 |
| 84 | CID_10648063 | 470.68 | 3.86 | 2 | 4 | 10 |
| 85 | [CID_11372643](https://pubchem.ncbi.nlm.nih.gov/compound/11372643) | 388.37 | 1.9 | 5 | 10 | 6 |
| 86 | CID_14589106 | 328.36 | 0.16 | 5 | 7 | 4 |
| 87 | CID_2694932 | 369.41 | 1.67 | 3 | 4 | 10 |
| 88 | CID_9064 | 290.27 | 1.33 | 5 | 6 | 1 |
| 89 | CID_5280961 | 270.24 | 1.91 | 3 | 5 | 1 |
| 90 | CID_5321859 | 346.29 | 2.28 | 4 | 8 | 3 |
| 91 | CID_5496475 | 360.31 | 3.03 | 3 | 8 | 4 |
| 92 | CID_15222911 | 344.32 | 3.22 | 2 | 7 | 4 |
| 93 | CID_493376 | 372.37 | 3.71 | 0 | 7 | 6 |
| 94 | CID_10787 | 126.11 | 0.83 | 3 | 3 | 0 |
| 95 | CID_7121 | 182.17 | 1.65 | 1 | 4 | 3 |
| **No.** | **Compound** | **MW** | **LogP** | **Hdon** | **Hacc** | **Rbon** |
| 96 | CID_370 | 170.12 | 0.21 | 4 | 5 | 1 |
| 97 | CID_3220 | 270.24 | 1.8 | 3 | 5 | 0 |
| 98 | CID_10639 | 284.26 | 2.45 | 2 | 5 | 1 |
| 99 | [CID_3082301](https://pubchem.ncbi.nlm.nih.gov/compound/3082301) | 129.16 | -1.5 | 4 | 5 | 2 |
| 100 | [CID_24721095](https://pubchem.ncbi.nlm.nih.gov/compound/24721095) | 398.44 | 3.2 | 2 | 6 | 6 |
| 101 | CID_45273405 | 350.41 | 4.1 | 1 | 5 | 5 |
| 102 | CID_5281233 | 314.47 | 6.9 | 1 | 2 | 5 |
| 103 | CID_5280805 | 610.52 | -1.4 | 10 | 16 | 6 |
| 104 | CID_12315350 | 354.36 | 3.8 | 1 | 6 | 5 |
| 105 | CID_443354 | 180.16 | -0.1 | 1 | 5 | 0 |
| 106 | CID_6325021c | 324.37 | 3.5 | 2 | 5 | 5 |
| 107 | [CID_15944778](https://pubchem.ncbi.nlm.nih.gov/compound/15944778) | 380.44 | 4.3 | 1 | 5 | 6 |
| 108 | [CID_5318767](https://pubchem.ncbi.nlm.nih.gov/compound/5318767) | 320.38 | 3.9 | 1 | 5 | 5 |
| 109 | CID_84298 | 244.29 | 2.7 | 2 | 4 | 4 |
| 110 | [CID_442433](https://pubchem.ncbi.nlm.nih.gov/compound/442433) | 194.19 | 0.7 | 0 | 6 | 0 |
| 111 | [CID_9940690](https://pubchem.ncbi.nlm.nih.gov/compound/9940690) | 366.44 | 4 | 1 | 5 | 6 |
| 112 | [CID_6325269](https://pubchem.ncbi.nlm.nih.gov/compound/6325269) | 338.4 | 3.6 | 1 | 6 | 5 |
| 113 | [CID_13892722](https://pubchem.ncbi.nlm.nih.gov/compound/13892722) | 392.46 | 4.2 | 1 | 6 | 6 |
| 114 | [CID_44583818](https://pubchem.ncbi.nlm.nih.gov/compound/44583818) | 447.53 | 3.5 | 1 | 8 | 5 |
| 115 | [CID_101439411](https://pubchem.ncbi.nlm.nih.gov/compound/101439411) | 478.52 | 4.8 | 1 | 7 | 7 |
| 116 | CID_118701382 | 504.55 | 5.1 | 1 | 7 | 7 |
| 117 | CID_102110701 | 490.5 | 4.9 | 1 | 8 | 7 |
| 118 | [CID_46173850c](https://pubchem.ncbi.nlm.nih.gov/compound/46173850) | 432.49 | 4.5 | 1 | 7 | 6 |
| 119 | [CID_131751540](https://pubchem.ncbi.nlm.nih.gov/compound/131751540) | 518.57 | 5.2 | 1 | 8 | 8 |
| **No.** | **Compound** | **MW** | **LogP** | **Hdon** | **Hacc** | **Rbon** |
| 120 | CID_5281643 | 270.24 | 2.1 | 3 | 5 | 2 |
| 121 | CID_10461942 | 406.45 | 4.4 | 1 | 6 | 6 |
| 122 | CID_11948668 | 364.43 | 4 | 1 | 6 | 5 |
| 123 | CID_21633105 | 452.51 | 4.7 | 1 | 7 | 7 |
| 124 | CID_101606290 | 496.54 | 5 | 1 | 8 | 8 |

**Table S2**: Differentiated Compounds between GF and GFC in Positive Ion Mode

| **No.** | **RT (min)** | **Formula** | **m/z actual value** | **Mass Error (ppm)** | | **m/z theoretical value** | **Description** |
| --- | --- | --- | --- | --- | --- | --- | --- |
| 1 | 3.98 | C_17_H_24_O_10_ | 411.1272 | 2.6661 | 149.0598; 209.0803; 249.0731; 411.1281 | | Geniposide |
| 2 | 4.28 | C_11_H_14_O_5_ | 249.0736 | 0.9600 | 163.0756; 209.0807; 249.0741; 265.0461; 353.1581 | | Genipin |
| 3 | 5.13 | C_11_H_10_O_4_ | 207.0645 | -3.0988 | 135.0434; 175.0380; 192.0430; 207.0640 | | Scoparone |
| 4 | 8.64 | C_10_H_14_O | 151.1114 | -2.3486 | 81.0712; 95.0499; 109.1016; 151.1113 | | Safranal |
| **No.** | **Retention time (min)** | **Formula** | **m/z actual value** | **Mass Error (ppm)** | **m/z theoretical value** | | **Description** |
| 5 | 10.02 | C_30_H_44_O_6_ | 501.3210 | -0.1895 | 465.2949; 501.3206; 523.3035 | | 10.02_500.3137n |
| 6 | 10.98 | C_30_H_48_O_6_ | 527.3330 | -2.4983 | 495.3081; 511.3386; 527.3325 | | Myrianthic acid |
| 7 | 12.25 | C_22_H_44_O_2_ | 358.3683 | 0.9983 | 340.3567; 358.3683 | | Behenic Acid |
| 8 | 13.41 | C_18_H_30_O_2_ | 279.2325 | 2.1834 | 125.0955; 135.1167; 195.1363; 209.1555; 223.1682; 261.2214; 279.2326 | | Linolenic acid |
| 9 | 14.12 | C_30_H_48_O_4_ | 495.3444 | -0.1938 | 455.3519; 495.3458 | | Siaresinol |
| **No.** | **Retention time (min)** | **Formula** | **m/z actual value** | **Mass Error (ppm)** | **m/z theoretical value** | | **Description** |
| 10 | 15.02 | C_30_H_48_O_5_ | 511.3378 | -3.1847 | 453.3380; 511.3419 | | Rotundic acid |
| 11 | 15.51 | C_27_H_28_O_13_ | 599.1163 | 0.1856 | 237.0736; 599.1168 | | 5-O-caffeoyl-4-O-sinapoylquinic acid |
| 12 | 15.97 | C_20_H_38_O_2_ | 328.3216 | 1.9240 | 311.2935; 328.3219 | | Ethyl oleate |

**Table S3**: Differentiated Compounds between GF and GFC in Negative Ion Mode

| **No.** | **Retention time (min)** | **Formula** | **m/z actual value** | **Mass Error (ppm)** | **m/z theoretical value** | **Description** |
| --- | --- | --- | --- | --- | --- | --- |
| 1 | 3.97 | C_16_H_22_O_10_ | 355.1041 | 1.6955 | 225.079 | Geniposidic acid |
| 2 | 5.12 | C_17_H_28_O_8_ | 395.1473 | -1.4867 | 89.0254; 102.9583; 135.0825; 395.1494 | 5.12_360.1779n |
| 3 | 5.42 | C_19_H_26_O_11_ | 465.1168 | -0.2566 | 61.9920; 157.0130; 207.0662 | 6'-O-acetylgeniposide |
| 4 | 5.74 | C_16_H_12_O_6_ | 335.0325 | -1.0013 | 199.0979 | 3-Methylkempferol |
| 5 | 7.77 | C_23_H_34_O_15_ | 549.1831 | 1.1853 | 453.3394 | GBGB |
| 6 | 8.32 | C_10_H_16_O_3_ | 165.0919 | -1.2337 | 105.0713; 149.0607; 165.0919 | Jasminodiol |
| 7 | 10.26 | C_20_H_24_O_4_ | 327.1614 | 3.6028 | 283.1711； 327.1610； 349.1431； 655.3253 | Crocetin |
| **No.** | **Retention time (min)** | **Formula** | **m/z actual value** | **Mass Error (ppm)** | **m/z theoretical value** | **Description** |
| 8 | 10.36 | C_19_H_18_O_7_ | 403.1045 | 2.9753 | 209.0463 | Gardenin B |
| 9 | 10.92 | C_30_H_44_O_6_ | 499.3073 | 1.6344 | 385.2403; 471.3130; 499.3059 | 10.92_499.3073m/z |
| 10 | 14.63 | C_30_H_46_O_4_ | 939.6721 | 0.1780 | 877.6355 | Gypsogenin |
| 11 | 14.93 | C_30_H_48_O_3_ | 455.3545 | 3.1097 | 407.3297; 455.3578 | 3-Epioleanolic acid |

**Table S4**: Linear Equations, Determinant coefficient (**R**^2^)，Ranges, Quantification Limits, and Detection Limits for the Quantification of 10 Differentiated Compounds in GF and GFC Extracts

| **No.** | **Compound** | **Linear Equation** | **R^2^** | **Linear Range（μg/mL）** | **LOQ（μg/mL）** | **LOD（μg/mL）** |
| --- | --- | --- | --- | --- | --- | --- |
| 1 | 3-Methylkempferol | y = 2.5429x + 0.1805 | 0.9992 | 0.29-44.45 | 0.290 | 0.096 |
| 2 | Gardenin B | y = 1.6526x + 6.4226 | 0.9993 | 5.19-508.77 | 1.216 | 0.402 |
| 3 | GBGB | y = 1.4891x + 1.5224 | 0.9993 | 0.89-105.18 | 0.754 | 0.249 |
| 4 | Geniposide (GE) | y = 0.9675x + 5.9324 | 0.9993 | 4.26-359.12 | 1.702 | 0.561 |
| 5 | Geniposidic acid | y = 2.9148x - 1.1167 | 0.9994 | 2.41-319.41 | 0.450 | 0.148 |
| 6 | Genipin | y = 3.1283x + 0.0336 | 0.9998 | 0.93-102.68 | 0.259 | 0.086 |
| 7 | Crocetin (CR) | y = 1.6308x + 7.5517 | 0.9995 | 7.97-870.28 | 1.893 | 0.624 |
| 8 | Crocin | y = 2.7185x + 1.6706 | 0.9997 | 3.77-426.84 | 0.547 | 0.181 |
| 9 | Crocin II | y = 2.2184x + 1.3604 | 0.9997 | 3.26-372.17 | 1.076 | 0.355 |
| 10 | Crocin III | y = 1.6135x + 0.9576 | 0.9993 | 0.32-187.06 | 0.321 | 0.106 |

**Table S5**: Quantification Results of Differentiated Compounds in GF and GFC Extracts (n = 3)

| **Content (mg·100 g^-1^) ± RSD** | **GF** | **CFC** |
| --- | --- | --- |
| 3-Methylkempferol | 179.06 ± 15.46 | 111.65 ± 7.44 |
| Gardenin B | 1233.72 ± 210.14 | 152.73 ± 27.66 |
| GBGB | 1212.38 ± 47.28 | 980.64 ± 5.9 |
| Geniposide | 2588.81 ± 447.90 | 675.56 ± 94.37 |
| Geniposidic acid | 262.99 ± 15.08 | 1814.51 ± 504.43 |
| Genipin | 94.19 ± 37.35 | 313.53 ± 31.93 |
| Crocetin | 964.36 ± 264.99 | 5734.10 ±1822.96 |
| Crocin | 1313.74 ± 236.71 | 208.31 ± 65.41 |
| Crocin II | 482.80 ± 89.22 | 154.38 ± 32.30 |
| Crocin III | 329.85 ± 73.20 | 115.75 ± 8.87 |

Note: Values are expressed as means ± standard deviations, n = 3.
